# Group Differences and Similarities in Mental Representation Structure of Tennis Serve

**DOI:** 10.3389/fpsyg.2020.552676

**Published:** 2020-10-30

**Authors:** Michael Gromeier, Christopher Meier, Thomas Schack

**Affiliations:** ^1^Neurocognition and Action-Biomechanics – Research Group, Bielefeld University, Bielefeld, Germany; ^2^Faculty of Psychology and Sport Science, Sport and Education Research Group, Bielefeld University, Bielefeld, Germany; ^3^Center of Excellence “Cognitive Interaction Technology”, Bielefeld University, Bielefeld, Germany; ^4^Research Institute for Cognition and Robotics, Bielefeld University, Bielefeld, Germany

**Keywords:** mental representation, proximal-to-distal-sequence, overhead motion, tennis serve, motor learning

## Abstract

A large number of studies have examined expertise and gender-related differences in the mental representation of motor skills in different sports, like throwing technique in judo, front loop in sailing, and integration of routines in the mental movement representation in volleyball. Also, tactical behavioral studies were conducted in futsal and football. In addition, studies were also carried out to support the motor learning process through mental training in golf. The Structural Dimensional Analysis-Motoric (SDA-M) method was also used in the medical sector for the rehabilitation of stroke patients. So far, few studies have investigated differences in the mental representation of a specific motor skill by experienced athletes of other related sports. The goal of the present study is to examine group differences and similarities in the mental representation of the tennis serve between experienced tennis, badminton, and handball athletes as well as a control group without any sport experience. We want to assess the quality of mental representation of technical-related overhead motion task expertise. For this purpose, we used the SDA-M to measure the mental representation of the tennis serve of four different groups (tennis, badminton, and handball athletes and a group of novices). As expected, badminton and handball athletes showed functionally well-structured representations, which were similar to the structure of the group of tennis athletes. Novices showed an unstructured mental representation. These outcomes confirm the relationship between mental representation and performance in the development of overhead motion. Furthermore, the results emphasize the importance of mental representations as an essential developmental aspect in learning motor skills, especially in learning technical-related motor skills.

## Introduction

According to Grosser and Neumaier (1982, p. 8), athletic performances are based on different factors, like tactical skills (sensory-cognitive abilities), conditional abilities, psychological capacity (mental representation), external conditions (environment), and technical skills (coordinative skills). In all kinds of sports, technical skills are highly relevant to solve movement problems. Therefore, high-level sport-specific motor skills can be classified as an important performance-determining factor for achieving the highest motor performance (Martin, 1980 in Niesner and Ranzmeyer, 1996, p. 29). According to Schack and Bar-Eli (2007, p. 62), “the success of an individual athlete or a team is highly dependent on how well the essential techniques of the sport are applied and mastered.”

One of the most difficult fundamental motor skills is proximal-to-distal sequences (P-D sequence), like overhead motions, which can be observed in a large variety of motions of the upper limbs of humans in both everyday life and sport activities (Serrien and Baeyens, 2017). Examples for sport-related motions with evidence for a P-D sequence are striking in volleyball (Wagner et al., 2012), tennis serve (Bahamonde, 2000; Marshall and Elliott, 2000; Wagner et al., 2012), javelin (Whiting et al., 1991), baseball (Hong et al., 2001), and overhead throwing in handball (van den Tillaar and Ettema, 2009b; Wagner et al., 2012; Serrien et al., 2015).

The P D-sequence, like the tennis serve, is a complex motor skill based on the general motor pattern (GMP) of *overhead motion* (Wagner et al., 2014). A GMP controls a whole class of movements and is characterized by transversal features (invariants) and motion-specific variable features (parameters). The overhead motion is fundamental for tennis service motion as well as for badminton clear (badminton) and overhead throwing (handball). All three movements demonstrate approximately the same order of the innervations of muscles (i.e., sequencing), which is characterized by a P-D sequence (Wagner et al., 2012). According to Wagner et al. (2014, p. 345), the “equal order of the proximal-to-distal sequencing and similar angles in the acceleration phase suggest there is a general motor pattern in overarm movements.” Additionally, all motor skills show the same relative time and duration (i.e., relative timing) and use of power (i.e., relative forces), and according to a functional biomechanics approach, tennis service motion, badminton clear, and overhead throwing can be characterized by the same functional movements: pre-activation, strike, and final swing (Göhner, 1992, 1979; Wagner et al., 2012, 2014). In sum, these overhead movements are assigned to different types of sports, but the movement of the badminton overhead clear corresponds to a handball overhead throwing and has certain similarities to a tennis serve, especially in the upper-body kinematics between throwing and hitting a ball, with or without a racket (Boeckh-Behrens, 1983; Wagner et al., 2012, 2014;, p. 48).

The optimal performance of these P-D sequences requires precise mechanics that involve coordinated kinetic chains to develop, transfer, and regulate the forces that are needed to withstand the inherent demands of the task and to allow optimal performance (Kibler et al., 2013). The term kinetic chain refers to the mechanical system by which athletes accomplish complex motor tasks such as throwing, hitting, and tennis or badminton serving as a result of the integrated, multisegmented, sequential joint motion and muscle activation system. A coordinated kinetic chain allows optimal force and accuracy to be developed in the core which can then be efficiently transferred to the arm during throwing motions (Sciascia and Cromwell, 2012). To produce optimal speed and/or high accuracy at the distal end of a kinematic chain, the involved body segments have to order in a specific way (Kibler et al., 2013). To generate a high end-point velocity by accelerating and decelerating adjacent links, body segments have to be ordered from proximal to distal in a sequential and functional manner. In this regard, functional refers to the movement quality (flow, rhythm, coupling, and precision of movements), an optimal acceleration, and the transfer of energy between body parts and the functional movement phases. So, the acquisition of a P-D sequence requires the coordination of the whole body, and this complex movement is a high requirement, especially in the learning process of P-D-sequences. An important factor which affects the P-D sequence is a well-structured pattern in mental representation, which is essential for the motor control and learning of motor skills (Schack, 2010).

In this study the mental representations, including the representation units and the structural composition of these representations, are of main interest. Mental representations are the cognitive imagery of motor skills which are used to refer to memory-related structures and processes that allow an athlete to use their experience to improve their motor performances. Memory-related structures are all states and processes stored and accessible in the memory, and they are the basis of the movement execution (Schack, 2012).

The cognitive architecture of complex actions refers to the interplay of higher levels of mental and lower levels of sensory motor, control, and representational systems, with mental representations playing a key role in motor control and learning (Schack, 2004). The resulting biomechanical structure of movements is herein not considered independent of the sensory effects of the motor action but rather is a result of the interplay across levels of action organization and, thus, is linked directly to cognitive–perceptual representations of the action.

In numerous studies, mental representations of complex sport movements have been found to be dependent on expertise (e.g., Schack and Mechsner, 2006; Schütz et al., 2009; Land et al., 2013). Experts and well-trained athletes show well-structured patterns of perceptual cognitive concepts, the so-called basic action concepts (BACs), and therefore show a more functional structure in the process of motor skills (e.g., Schack and Mechsner, 2006; Bläsing et al., 2009; Stöckel et al., 2012). Basic action concepts contain anticipated movement effects and therefore contain integrating feature-based units that represent the functional, sensory, and spatio-temporal and biomechanical characteristics of a movement (Schack, 2010). By contrast, novices and less trained individuals usually showed unstructured mental representation to motor skills (e.g., Schack and Mechsner, 2006; Bläsing et al., 2009; Stöckel et al., 2012) during the learning process of motor skills (e.g., Frank et al., 2016). Schack and Mechsner (2006) investigated the mental representations of the tennis service motion for different expertise levels (high-level tennis experts, low-level athletes, and novices). The results highlighted that high-level tennis experts showed a well-structured mental representation, and these representational frameworks were organized in a hierarchical tree-like structure. A study of Meier et al. (2020) showed that mental representations of the tennis serve changed over time during skill acquisition with explicit or analogy instructions.

The transfer of motor learning was investigated by van den Tillaar and Ettema (2009a). When expert handball athletes had to throw with the dominant or non-dominant arm, the sequence of maximal velocities remained invariant, with changes in the timing of maximal angles (van den Tillaar and Ettema, 2009a). With only minor influences of other changing organismic constraints, it is shown that the biomechanical, neurological, and ontogenic constraints provide a strong predisposition for this P-D sequence (van den Tillaar and Ettema, 2009a).

Comparing the P-D sequence of different motor skills, studies have found both similarities (Broer and Houtz, 1967; Wagner et al., 2012) and differences (Adrian and Engberg, 1971; Anderson, 1979) in the movement pattern. Overall, it is shown that there is a main topological P-D sequence. A variable training intervention and an expertise in more than one motor skill with a P-D sequence probably favor the election of the P-D-sequence for a specific motor skill and lead to a better adaption to specific task constraints (Serrien and Baeyens, 2017).

With respect to mental representation, as an essential factor for motor control and learning, the question arises on whether mental representations may transfer across motor skills that involve a similar P-D sequence. So far, no studies have investigated differences in the mental representation of a specific motor skill by experienced athletes of other related sports.

The aim of the present study is to examine the tennis serve’s mental representation of experienced tennis, badminton, and handball athletes and novices as well as to analyze the differences and the similarities between their mental representation structures. The results of this study could benefit the process of motor learning of technique-related motor skills and could give valuable insights in a possible transfer of mental representations across experts from sports that require the completion of similar, yet distinct, P-D sequence tasks.

First, we assume that the tennis experts’ mental representation show the most functional links, whereas novices demonstrate an unstructured mental representation.

Second, we expect that experience in a specific P-D sequence (i.e., overhead throw and badminton clear) should also be accompanied by a functional structured representation of the technically related tennis serve. Thus, we hypothesize that the mental representations of badminton and handball athletes show no significant differences but differ from those of tennis experts and novices.

## Materials and Methods

### Participants

In this study, 40 participants were divided into four groups: tennis (*n* = 10; *M*_age_ = 29.9 years, SD_age_ = 8.5; four females, six males; training age = 24.1 years; training frequency = 9.4 h per week); badminton (*n* = 10; *M*_age_ = 28.5 years, SD_age_ = 5.9; five females, five males; training age = 19.2 years; training frequency = 5.4 h per week); handball (*n* = 10; *M*_age_ = 27.6 years, SD_age_ = 4.0; six females, four males; training age = 21.2 years; training frequency = 3.8 h per week); or novices (*n* = 10; *M*_age_ = 30.3 years, SD_age_ = 4.8; five females, five males) without prior sport experience in tennis, badminton, or handball.

At the time of examination, all athletes (tennis, badminton, and handball athletes) were members of sports clubs and active athletes. Throughout the testing, all participants were healthy and in good condition. To take part in the experiment, the participants gave informed consent. The study involving human participants was approved by the Ethics Committee of Bielefeld University and adhered to the ethical standards of the latest revision of the Declaration of Helsinki.

### Analysis of Mental Representation Structure

The storage of motor skills’ mental representation can be assessed using the Structural Dimensional Analysis-Motoric (SDA-M; Schack, 2004) method. Based on the approach of cognitive architecture of human motion (Lander and Lange, 1996; Schack, 2012, 2004), the SDA-M objectifies the cognitive representations of movements in long-term memory.

The SDA-M method comprises four steps (Schack, 2012): In the first step, the split procedure (described in more detail below), delivering a distance scaling between the BACs of a suitably pre-determined set, is conducted. In the second step of SDA-M, a hierarchical cluster analysis is used to transform each set of BACs into a hierarchical structure (depicted as a dendrogram). In the third step, a factor analysis reveals the dimensions in this structured set of BACs. In the last step, a within- and between-group comparison of the cluster solutions was performed by determining the structural invariance between cluster solutions.

### Stimulus

The tennis serve can be subdivided into three distinct biomechanical and functional phases (Göhner, 1992, 1999), namely, pre-activation (i.e., best possible prerequisites for energy production are generated), strike (i.e., energy is transferred), and final swing (i.e., movement and racket are decelerated). In the present study, we used a pre-determined set of 11 BACs [based on a study by Schack and Mechsner (2006)] of the tennis serve (Table 1). For each phase, Schack and Mechsner (2006) determined a set of BACs solving a biomechanical subproblem of the overall movement: BAC 1–4 for pre-activation, BAC 5–8 for strike, and BAC 9–11 for final swing.

**TABLE 1:** Basic action concepts and movement phases of the tennis serve.

**No.**	**Basic action concept (BAC)**	**Movement phase (clusters 1–3)**
1	Throwing ball	Pre-activation (1)
2	Putting forward	Pre-activation (1)
3	Bending knee	Pre-activation (1)
4	Bending elbow	Pre-activation (1)
5	Turning up body	Strike (2)
6	Racket acceleration	Strike (2)
7	Stretching whole body	Strike (2)
8	Hitting ball	Strike (2)
9	Wrist fold down	Final swing (3)
10	Racket swing out	Final swing (3)
11	Slowing down	Final swing (3)

*Each of the BACs can be functionally assigned to one particular movement phase of the tennis serve (Schack and Mechsner, 2006, p. 79).*

**FIGURE 1:**
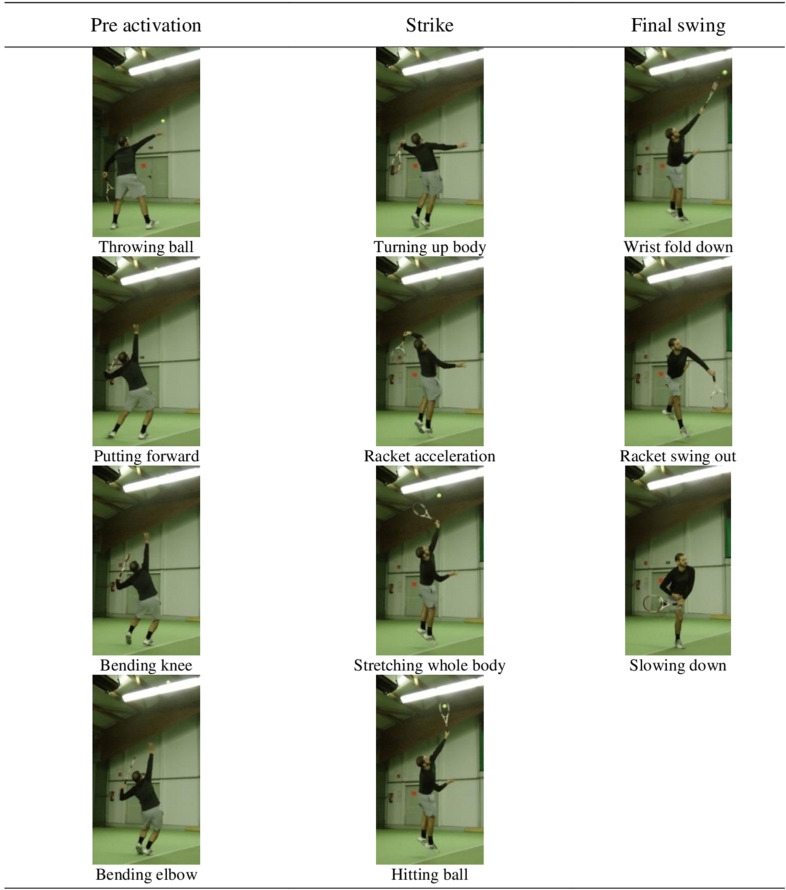
The images, with the term below used, represent the basic action concepts of the tennis serve.

The display format of the BACs was adapted from Hennig et al. (2017), providing a combination of image and text items. Therefore, we extracted an image with the software *HD Writer AE 1.2* for each BAC from a video showing the tennis serve’s movement in slow motion. In order to ensure that the images correspond to the respective textual descriptions, the allocation was validated by two experienced tennis experts (coaches with B-/A-level) who did not take part in the study. In this way, it has been possible to provide clear and adequate stimulus material combining images and textual descriptions (Figure 1). The participants match to the “model” portrayed in the images in the way that they are nearly in the same age, about 25. In order to avoid ambiguities, we provide a combination of images and textual descriptions. The use of text or combined items is recommended when applying the SDA-M to children, adolescents, or adults (Schack, 2012).

### Split Procedure

In order to determine relations between BACs and groupings of BACs, a split procedure was conducted (i.e., first step of the SDA-M). The participants sat approximately 60 cm away from a 15-in. laptop screen. The stimuli had a size of 9.0–9.0 cm (250–250 pixels) and were shown on a laptop (Sony Vaio). Each BAC (i.e., image plus textual description) was presented in an anchor position, which was displayed in the upper half of the laptop screen. The remaining BACs were successively displayed below them. For each pair of BACs (i.e., anchor BAC and one additional BAC), the participants were asked to decide whether or not the BACs presented on the screen belonged together during movement execution. Once all BACs had been assessed in relation to the anchor BAC, a different BAC took over the anchor position, and the procedure was repeated. The split procedure ended after all BACs had been in the anchor position (Schack, 2012; Meier et al., 2020). All participants were individually tested, without any time pressure. The testing took 25 min.

### Data Analysis

Mental representation structures were analyzed by determining mean group dendrograms *via* cluster analyses (Schack, 2012). The red horizontal dashed line indicates the critical value (*d*_crit_ = 3.51) for a significant alpha-level of *p* = 0.05. Statistically, all BACs, which form structures below the critical value, are clustered together. The lower the value of a link between two items, the shorter the distance is between the related BACs.

Analyses of invariance were performed to compare differences between clusters. The final comparison of *λ* with *λ*_crit_ = 0.68 makes statements about the manner, in which the cluster solutions have a significantly similar structure to a predefined reference (*λ*_ideal_ = 1). This means that there was a significant difference between clusters if *λ* < *λ*_crit_ = 0.68. If *λ* is greater than *λ*_crit_, there is a significant similarity, which corresponds to an alpha level of 5% (Schack, 2012). Moreover, the Adjusted Rand Index (ARI) ranks the similarity of the groups’ mental representation (Santos and Embrechts, 2009). The ARI ranges as an index of similarity from -1 to 1.

## Results

### Within-Group Comparisons

**FIGURE 2:**
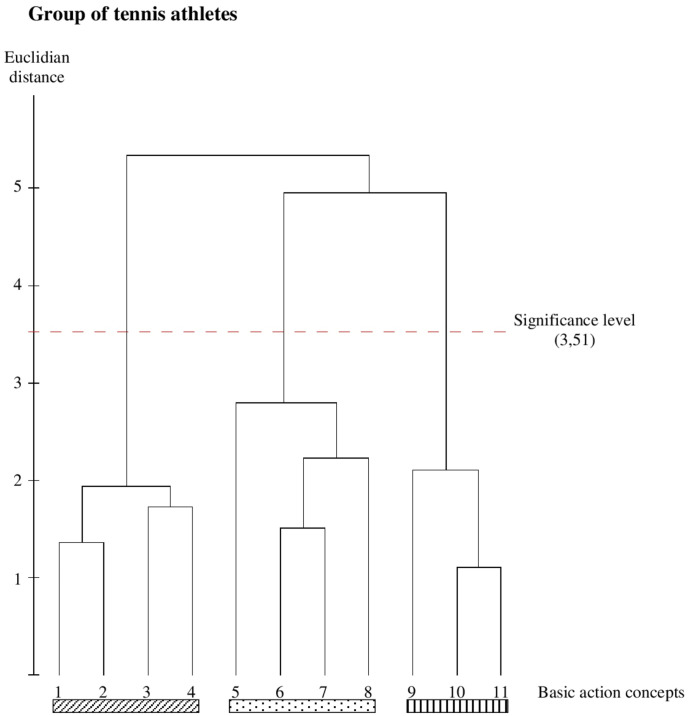
Outcome of the hierarchical cluster analysis for the tennis group (*n* = 10) displayed as dendrogram (*p* = 5%; cleth = 3.51). The BACs are marked with numbers below dendogram. The resulting clusters are highlighted with differentbars (pictures 1–4, pre activation BACs, diagonally striped bar; pictures 5–8, strike BACs, dot bar; picture 9–11, final swing BACs, vertically striped bar).

**FIGURE 3:**
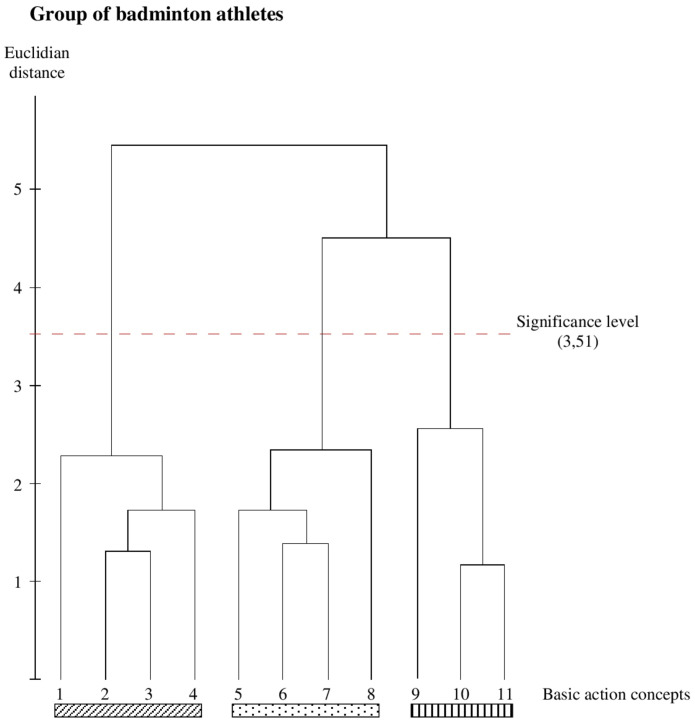
Outcome of the hierarchical cluster analysis for the badminton group (*n* = 10) displayed as dendogram (*p* = 5%; d_crit_ = 3.51). The BACs are marked with numbers below dendogram. The resulting clusters are highlighted with different rectangle (pictures 1–4, pre activation BACs, diagonally striped bar; pictures 5–8, strike BACs, dot bar; picture 9–11, final swing BACs, vertically striped bar).

Both the tennis athletes’ (Figure 2) and the badminton athletes’ (Figure 3) mean dendrograms reveal the optimal cluster solution of the SDA-M and show a clear separation of the three functional movement phases. More specifically, the first cluster marked with a diagonally striped bar represents all BACs of the pre-activation phase: BAC 1 (throwing ball), BAC 2 (putting forward), BAC 3 (bending knee), and BAC 4 (bending elbow). The second cluster marked with a dot bar is related to the strike phase with BAC 5 (turning up body), BAC 6 (racket acceleration), BAC 7 (stretching whole body), and BAC 8 (hitting ball). The third cluster marked with a vertically striped bar includes BAC 9 (wrist fold down), BAC 10 (racket swing out), and BAC 11 (slowing down), representing all BACs of the final swing phase.

**FIGURE 4:**
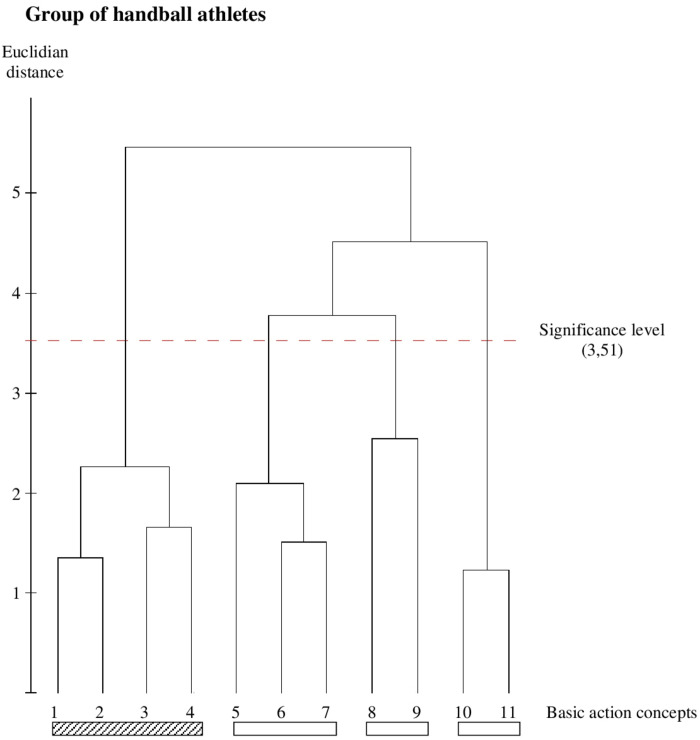
Outcome of the hierarchical cluster analysis for the badminton group (*n* = 10) displayed as dendogram (*p* = 5%; d_crit_ = 3.51). The BACs are marked with numbers below dendogram. The resulting clusters are highlighted with different rectangle (pictures 1–4, pre activation BACs, diagonally striped bar; pictures 5–7, 8–9, 10–11, three unstructured clusters, blank bar).

The handball athletes’ dendrogram (Figure 4) shows a well-structured representation in the pre-activation phase (BACs 1–4), but not in the strike and the final swing phase. In cluster 2 relating to the strike phase, BAC 8 is missing and connected with BAC 9 and thus forms its own third cluster. Lastly, the fourth cluster consists of BAC 10 and BAC 11 representing two of the three BACs of the final swing phase.

**FIGURE 5:**
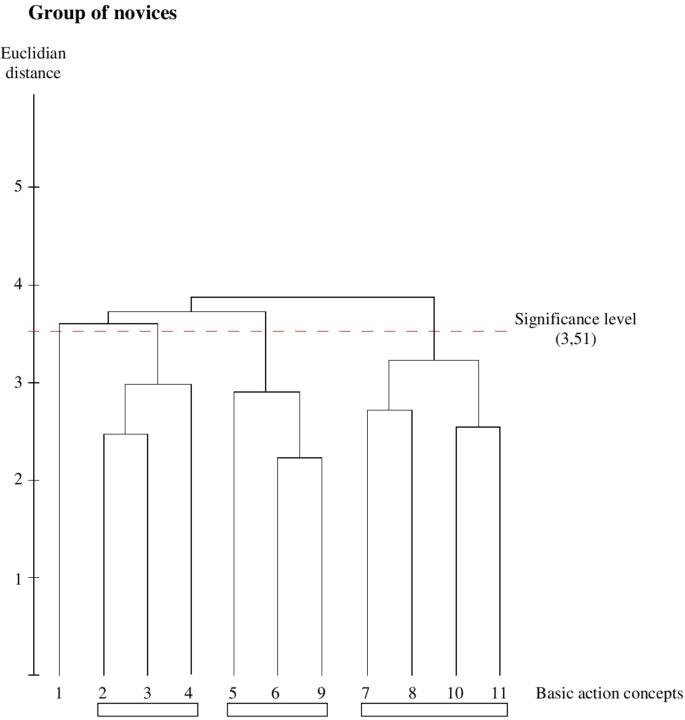
Outcome of the hierarchical cluster analysis for the group of novices (*n* = 10) displayed as dendogram (*p* = 5%; &Tit = 3.51). The BACs are marked with numbers below dendogram. The resulting clusters are highlighted with different rectangle (three unstructured clusters, blank bar).

The novices’ dendrogram (Figure 5) demonstrates three separated clusters. The first cluster is grouped by BAC 2, 3, and 4, which corresponds to the pre-activation phase. The second cluster, actually the strike phase, includes BAC 5, 6, and 9. BAC 7, as well as BAC 8, is assigned to the third cluster consisting of BACs 7, 8, 10, and 11. Moreover, BAC 9 follows the racket acceleration (BAC 6) and not hitting the ball (BAC 8).

### Between-Group Comparisons

As can be seen in Figures 1, 2, both the tennis group and the badminton group show identical functional clusters, resulting in an invariant representation structure (*λ* = 1.0), whereas both groups significantly differ from the handball group (*λ* = 0.468 for both) as well as from the group of novices (*λ* = 0.565 for both). Additionally, statistical analysis of invariance demonstrate a significant difference between the handball group and the novice’s group (*λ* = 0.457).

The ARI indicates that the mental representation structure of the tennis group is identical to the mental representation of the badminton group (ARI = 1.0) as well as less similar to the handball group (ARI = 0.7) and the group of novices (ARI = 0.266). The comparisons of the badminton group with the handball group as well as with the novices group demonstrate the same results (ARI = 0.7 for the comparison with the handball group and ARI = 0.266 for the comparison with the novice’s group). The handball group and the novices group vary widely (ARI = 0.285).

## Discussion

The aim of this study was to examine the tennis serve’s mental representation of experienced tennis, badminton, and handball athletes and novices as well as to analyze the differences and the similarities between their mental representation structures. We assume that the tennis experts’ mental representation shows the most functional links, whereas novices demonstrate an unstructured mental representation. Furthermore, we expect that experience in a specific P-D sequence (i.e., overhead throw and badminton clear) should also be accompanied by a functional structured representation of the technically related P-D sequence (tennis serve). Thus, we hypothesize that the mental representations of badminton and handball athletes show no significant differences but differ from those of tennis experts and novices.

The outcome of the hierarchical cluster analysis of tennis athletes is very much in line with the biomechanical functional structure of the tennis service motion according to Schack and Mechsner (2006), both in temporally and functionally aspects. Therefore, the dendrogram represents the optimal cluster solution of the SDA-M. The results highlighted a high degree of order formation in the specific movement knowledge of the tennis athletes. The hierarchical cluster analysis of the grouping diagram of the badminton athletes provides identical results as the group of the tennis athletes. The chronological and the functional aspects of the hierarchical representation structure also correspond to the biomechanical structure of tennis serve. The badminton athletes show a clear structure in their mental representation so that there are three significant clusters. Their cluster solution corresponds to the mental representation structure of the tennis athletes. The results highlighted that tennis and badminton athletes showed a well-structured mental representation, and these representational frameworks were organized in an optimal hierarchical tree-like structure. So, mental representation is structured in exactly the same way the movement is organized. Both the tennis serve and the badminton clear consist of three distinct functional movement phases: pre-activation (first auxiliary function phase), strike (main function phase), and final swing (second auxiliary function phase). As expected, experiences in badminton clear are accompanied by a developed structure of the technical-related tennis serve. This is confirmed by the studies of Wagner et al. (2014, 2012) and Broer and Houtz (1967) who found similarities in the P-D sequence of different overhead motions. Therefore, it was shown that there is a main topological P-D sequence in the overhead motions (Göhner, 1992, 1999; Serrien and Baeyens, 2017).

Although the movement of the badminton clear corresponds to an overhead throwing movement in handball and has certain similarities to tennis serve (Boeckh-Behrens, 1983; Göhner, 1992, 1979), the handball athletes show a problematic structure in their mental representation. The cluster solutions highlighted differences in the mental representations of handball athletes. The outcome of the hierarchical cluster analysis of handball athletes shows a well-structured movement representation in the pre-activation, but not in strike and final swing. The group of handball athletes has problems to assign the end of the strike and the short period of hitting ball (BAC 8). As a result, the timing of the hitting ball is wrongly represented.

The group of novices, the group without any comparable sporting experience, shows an unstructured movement representation in their movement memory. With regard to the classification of the single BACs to the corresponding function phases, the participants of group novices obviously achieve fewer good results than the other three groups. Thus, no function phase is structured by any of the participants in an optimal manner. In contrast to the group of tennis, badminton, and handball athletes, the mental representations of novices were organized less hierarchically, were more variable among persons, and were less well-matched with functional and biomechanical demands, which are in line with the studies of Schack and Mechsner (2006) and Bläsing et al. (2009).

Reasons for the similarity in the cluster solution of tennis and badminton athletes can be found in the certain degree of similarity of the analyzed motor skill. The main function phase consists of the forearm and humerus actions, and the racket movement starts with the stroke and ends with the ball leaving the racket. The final swing can be characterized as the second auxiliary function phase, and it is used to slow down the forward movement and control the swing out of the main function phase which enormously accelerated the racket. In tennis and badminton, the racket position and aspects like the velocity, swing, and orientation of the racket have to be controlled. These racket performances, especially hitting the ball with the racket, are key aspects for movements characteristic and specific to racket sports, like tennis, badminton, squash, and table tennis. Tennis and badminton athletes have to hit the ball, whereas a handball player already holds the ball in the hand when throwing. In handball, the forearm and humerus actions, which form the forearm whip, are the most important component in the overarm throwing movement (Gromeier et al., 2017). The obvious differences between tennis, badminton, and handball athletes are mainly due to the main function phase, more precisely in *hitting the ball* (BAC 8), which is not necessary in handball. BACs 5 to 7 (turning up body, racket acceleration, and stretching whole body) are well structured and correspond to the biomechanical structure of tennis serve.

So, reasons for the different cluster solutions of tennis, badminton, and handball group could be found in the use of specific sport equipment, i.e., racket in tennis and badminton, and the high requirement in coordination of all body segments to produce high accuracy at the distal end of a P-D sequence (Kibler et al., 2013). That underlies the results of Wagner et al. (2014), who found an identical order of movement phase between different overhead motions but significant differences in timing. Obviously, the throwing situation in handball competitions is very different from situations in tennis and badminton competitions. In handball, there is a huge variability, and the variety of situations is influenced by opponents and teammates, which leads to different execution conditions. So, another reason for different cluster solutions may be caused by the various real game situations.

## Conclusion

We conclude that the mental representations of the tennis serve in tennis, badminton, and handball athletes are quite similar but not identical. This may be due to specific adaptations based on technical and tactical requirements in different sport disciplines and, in particular, the different upper body movements and the use of a racket (Wagner et al., 2014, p. 353). However, despite some differences, a nearly uniform pattern can be seen and all cluster solutions are characterized by the same functional components: pre-activation, strike, and final swing. Therefore, we suggest that the overhead motion is reflected in the mental representation structure of badminton and handball players.

The overhead motion is an important part of the integrative (non-specific) concept of team ball games according to the game implicit learning model of Roth et al. (2002). Therefore, it is an important part of physical education. Among throwing games, the technically correct throwing movement is relevant for and comparable to many sport disciplines such as handball and tennis, yet it is also fundamental for learning the process of the javelin throw. Transferring this assumption to learning processes, an improvement in the mental representations of a basic motor skill, like *overhead motion*, may be accompanied by benefits in mental representations of related motor skills (e.g., overhead throwing motion) and therefore has positive effects in the quality of movement execution. This approach might also be conceivable in the opposite direction. Accordingly, developments in the mental representation of a specific motor skill (e.g., badminton clear) may benefit the mental representations to related motor skills (e.g., overhead motion), which could be linked with qualitative and quantitative development in performance.

This could provide important information to understand the transfer of mental representations and, therefore, the transfer of motor skills in the motor learning process and to supply an approach that is useful to practice and alter different overhead motions, like overhead throwing movement (Wagner et al., 2012).

Indeed it has been shown that a motor skill with relatively complex movement dynamics can be acquired implicitly, without the accrual of consciously accessible declarative knowledge and without a corresponding increase in verbal knowledge about the motor skill (Steenbergen et al., 2010). This may eventually enhance the opportunities for action of individuals with movement problems. The results emphasize the importance of mental representations as an essential developmental aspect in learning motor skill movement techniques, being obsessed in transferring these mental representations and learning technical-related motor skill movement technique. This might be a beneficial approach to come up given the difficulties in the learning process of the overhead throwing movement. Published studies point to gender-related differences in the qualitative throwing movement characteristics in favor of male novices (Thomas and French, 1985; Winter, 1987; Nelson et al., 1991; Goodway and Lorson, 2008; Lorson et al., 2013; Gromeier et al., 2019). Also, age-related differences were analyzed (Halverson et al., 1982; Winter, 1987; Lorson et al., 2013; Gromeier et al., 2019). To optimize technical and mental training in specific overhead motions and therefore to improve the sports performances of novices and athletes, physical and university education should support a wide development in addressing basic motor skills and practice basic movement pattern needed to achieve specific nodes of action. A mental training and developing mental representation of basic movement pattern with P-D sequences probably favor the transfer and the election of the P-D sequence for a related motor skill. That also could lead to a better adaption to specific movement tasks with P-D sequence. Another important point is that, in the basic training of physical education, there is no inevitable need for expensive equipment of tennis or badminton, which helps schools to minimize this cost factor.

Therefore, it remains to be shown how far the improvement of such basic skills is accompanied by an improvement of related motor performance. The results are also limited by the fact that the present study examined only the mental representation of the tennis serve and not the overhead throwing movement in handball or badminton clear. Further studies should perform motoric tests to investigate qualitative and quantitative data of different overhead motions. Another interesting avenue for further research that could benefit our understanding is how the P-D sequence evolves during learning in a new field of sport. To do so, studies should focus on learning a P-D sequence of different types of motion (e.g., overhead throwing) and examine how this affects the outcome of the actual P-D sequence (e.g., tennis serve).

## Data Availability Statement

The raw data supporting the conclusions of this article will be made available by the authors, without undue reservation.

## Ethics Statement

Ethical review and approval was not required for the study on human participants in accordance with the local legislation and institutional requirements. The ethics committee waived the requirement of written informed consent for participation.

## Author Contributions

CM and MG contributed to the conception and design of the study and performed the statistical analysis. MG wrote the first draft of the manuscript. All authors wrote sections of the manuscript and contributed to manuscript revision, read and approved the submitted version.

## Conflict of Interest

The authors declare that the research was conducted in the absence of any commercial or financial relationships that could be construed as a potential conflict of interest.

## References

[B1] Adrian M. J. Engberg M. L. (1971). “Sequential timing of three overhead patterns,” in *Kinesiology Review*, ed. Widule C. (Washington, DC: AAPHER).

[B2] Anderson M. B. (1979). Comparison of muscle patterning in the overarm throw and tennis serve. *Res. Q.* 50 541–553. 10.1080/00345377.1979.10615649

[B3] Bahamonde R. E. (2000). Changes in angular momentum during the tennis serve. *J. Sports Sci.* 18 579–592. 10.1080/02640410050082297 10972409

[B4] Bläsing B. Tenenbaum G. Schack T. (2009). The cognitive structure of movements in classical dance. *Psychol. Sport Exerc.* 10 350–360. 10.1016/j.psychsport.2008.10.001

[B5] Boeckh-Behrens W.-U. (1983). *Badminton Heute: Gründliche Einführung; Perfekte Technik; Erfolgreiche Taktik; Systematisches Training.* Krefeld: Intermedia.

[B6] Broer M. R. Houtz S. J. (1967). *Patterns of Muscular Activity in Selected Sports Skills.* Springfield, IL: Charles, C Thomas.

[B7] Frank C. Land W. M. Schack T. (2016). Perceptual-cognitive changes during motor learning: the influence of mental and physical practice on mental representation, gaze behavior, and performance of a complex action. *Front. Psychol.* 6:1981. 10.3389/fpsyg.2015.01981 26779089 PMC4705276

[B8] Göhner U. (1979). *Bewegungsanalyseim Sport.* Schorndorf: Verlag Karl Hofmann.

[B9] Göhner U. (1992). *Einführung in die Bewegungslehre des Sports, Teil 1: Die sportlichen Bewegungen.* Schorndorf: Verlag Karl Hoffmann.

[B10] Göhner U. (1999). *Einführung in die Bewegungslehre des Sports. Teil 2: Bewegerlehre des Sports.* Schorndorf: Hofmann.

[B11] Goodway J. D. Lorson K. M. (2008). Gender differences in throwing form of children ages 6-8 years during a throwing game. *Res. Q. Exerc. Sport* 79 174–182. 10.1080/02701367.2008.10599481 18664042

[B12] Gromeier M. Koester D. Schack T. (2017). Gender differences in motor skills of the overarm throw. *Front. Psychol.* 8:212. 10.3389/fpsyg.2017.00212 28261142 PMC5313487

[B13] Gromeier M. Schack T. Koester D. (2019). Does physical education foster skill acquisition in novices from childhood to adolescence? *Adv. Soc. Sci. Res. J.* 6 30–49.

[B14] Grosser M. Neumaier A. (1982). *Techniktraining. Theorie und Praxis Aller Sportarten.* München: BLV-Buchverlag.

[B15] Halverson L. E. Roberton M. A. Langendorfer S. (1982). Development of the overarm throw: movement and ball velocity changes by seventh grade. *Res. Q. Exerc. Sport* 53 198–205. 10.1080/02701367.1982.10609340

[B16] Hennig L. Ghesneh M. Mack M. Heinen T. (2017). Development of individual instructions based on pupils’ mental representations of a gymnastics skill. *J. Phys. Educ. Sport* 17 2604–2611.

[B17] Hong D. A. Cheung T. K. Roberts E. M. (2001). A three-dimensional, six-segment chain analysis of forceful overarm throwing. *J. Electromyogr. Kinesiol.* 11 95–112. 10.1016/s1050-6411(00)00045-6 11228423

[B18] Kibler W. B. Kuhn J. E. Wilk K. E. Sciascia A. Moore S. Laudner K. (2013). The disabled throwing shoulder: spectrum of pathology – 10-year update. *Arthroscopy* 29 141–161. 10.1016/j.arthro.2012.10.009 23276418

[B19] Land W. M. Volchenkov D. Bläsing B. Schack T. (2013). From action representation to action execution: exploring the links between cognitive and biomechanical levels of motor control. *Front. Comput. Neurosci.* 7:127. 10.3389/fncom.2013.00127 24065915 PMC3776155

[B20] Lander H.-J. Lange K. (1996). Untersuchung zur Struktur- und Dimensionsanalysebegrifflich repräsentierten Wissens. *Z. Psychol.* 204 55–74.

[B21] Lorson K. M. Stodden D. F. Langendorfer S. J. Goodway J. D. (2013). Age and gender differences in adolescent and adult overarm throwing. *Res. Q. Exerc. Sport* 84 239–244. 10.1080/02701367.2013.784841 23930550

[B22] Marshall R. N. Elliott B. C. (2000). Long-axis rotation: the missing link in proximal-to-distal segmental sequencing. *J. Sports Sci.* 18 247–254. 10.1080/026404100364983 10824641

[B23] Meier C. Frank C. Gröben B. Schack T. (2020). Verbal instructions and motor learning: how analogy and explicit instructions influence the development of mental representations and tennis serve performance. *Front. Psychol.* 11:2. 10.3389/fpsyg.2020.00002 32116881 PMC7019697

[B24] Nelson R. N. Thomas J. R. Nelson J. K. (1991). Longitudinal change in throwing perfor-mance. *Res. Q. Exerc. Sport* 62 105–108. 2028085 10.1080/02701367.1991.10607526

[B25] Niesner H. W. Ranzmeyer J. H. (1996). *Badminton. Reinbek.* Leipzig: Sportiv Badminton.

[B26] Roth K. Kröger C. Memmert D. (2002). *Ballschule Rückschlagspiele.* Schorndorf: Hofmann.

[B27] Santos J. Embrechts M. (2009). “On the use of the adjusted rand index as a metric for evaluating supervised classification,” in *Artificial Neural Networks – ICANN 2009. ICANN 2009. Lecture Notes in Computer Science*, eds Alippi C. Polycarpou M. Panayiotou C. Ellinas G. (Berlin: Springer), 175–184. 10.1007/978-3-642-04277-5_18

[B28] Schack T. (2004). The cognitive architecture of complex movement. *Int. J. Sport Exerc. Psychol.* 2 403–438. 10.1080/1612197x.2004.9671753

[B29] Schack T. (2010). *Die Kognitive Architektur Menschlicher Bewegungen: Innovative Zugänge zu Psychologie (1).* Aachen: Meyer and Meyer Sport.

[B30] Schack T. (2012). “Measuring mental representations,” in *Measurement in Sport and Exercise Psychology*, eds Tenenbaum G. Eklund R. C. Kamata A. (Champaign, IL: Human Kinetics), 203–214.

[B31] Schack T. Bar-Eli M. (2007). “Psychological factors in technical preparation,” in *Psychology of Sport Training*, eds Blumenstein B. Lidor R. (Münster: Meyer and Meyer), 62–103.

[B32] Schack T. Mechsner F. (2006). Representation of motor skills in human longterm memory. *Neurosci. Lett.* 391 77–81. 10.1016/j.neulet.2005.10.009 16266782

[B33] Schütz C. Klein-Soetebier T. Schack T. (2009). “Modeling of biomechanical parameters based on LTM structures,” in *Human Centered Robot Systems. Cognitive Systems Monographs*, eds Ritter H. Sagerer G. Dillmann R. Buss M. (Berlin: Springer), 161–171. 10.1007/978-3-642-10403-9_17

[B34] Sciascia A. Cromwell R. (2012). Kinetic chain rehabilitation: a theoretical framework. *Rehabil. Res. Pract.* 4 1–9. 10.1155/2012/853037 22666599 PMC3361354

[B35] Serrien B. Baeyens J.-P. (2017). The proximal-to-distal sequence in upper-limb motions on multiple levels and time scales. *Hum. Mov. Sci.* 55 156–171. 10.1016/j.humov.2017.08.009 28837899

[B36] Serrien B. Clijsen R. Blondeel J. Goossens M. Baeyens J. P. (2015). Differences in ball speed and three-dimensional kinematics between male and female handball players during a standing throw with run-up. *BMC Sports Sci. Med. Rehabil.* 7:27. 10.1186/s13102-015-0021-x 26587236 PMC4652456

[B37] Steenbergen B. van der Kamp J. Verneau M. Jongbloed-Pereboom M. Masters R. (2010). Implicit and explicit learning: applications from basic research to sports for individuals with impaired movement dynamics. *Disabil. Rehabil.* 32 1509–1516. 10.3109/09638288.2010.497035 20575752

[B38] Stöckel T. Hughes C. Schack T. (2012). Representation of grasp postures and anticipatory motor planning in children. *Psychol. Res.* 76 768–776. 10.1007/s00426-011-0387-7 22075763

[B39] Thomas J. R. French E. (1985). Gender Differences across age in motor performance – A meta-analysis. *Psychol. Bull.* 98 269–282. 3901062

[B40] van den Tillaar R. Ettema G. (2009a). A comparison of overarm throwing with the dominant and nondominant arm in experienced team handball players. *Percept. Mot. Skills* 109 315–326. 10.2466/pms.109.1.315-326 19831111

[B41] van den Tillaar R. Ettema G. (2009b). Is there a proximal-to-distal sequence in overarm throwing in team handball? *J. Sports Sci.* 27 949–955. 10.1080/02640410902960502 19629844

[B42] Wagner H. Pfusterschmied J. Tilp M. Landlinger J. von Duvillard S. P. Muller E. (2014). Upper-body kinematics in team-handball throw, tennis serve, and volleyball spike. *Scand. J. Med. Sci. Sports* 24 345–354. 10.1111/j.1600-0838.2012.01503.x 22813080

[B43] Wagner H. Pfusterschmied J. von Duvillard S. Müller E. (2012). Skill-dependent proximal-to-distal sequence in team-handball throwing. *J. Sports Sci.* 30 21–29. 10.1080/02640414.2011.617773 22111879

[B44] Whiting W. C. Gregor R. J. Halushka M. (1991). Body segment and release parameter contributions to new-rules javelin throwing. *Int. J. Sport Biomech.* 7 111–124. 10.1123/ijsb.7.2.111

[B45] Winter R. (1987). “Die motorische Entwicklung des Menschen von der Geburt bis ins hohe Alter (Überblick),” in *Bewegungslehre – Sportmotorik*, eds Meinel K. Schnabel G. (Berlin: Sportverlag), 275–397.

